# Postnatal corticosteroids and developmental outcomes in extremely preterm or extremely low birth weight infants: The Victorian Infant Collaborative Study 2016–17 cohort

**DOI:** 10.1111/apa.16696

**Published:** 2023-02-15

**Authors:** Ellen Douglas, Kate A. Hodgson, Joy E. Olsen, Brett J. Manley, Calum T. Roberts, Elisha Josev, Peter J. Anderson, Lex W. Doyle, Peter G. Davis, Jeanie L. Y. Cheong, the Victorian Infant Collaborative Study Group, Rosemarie Boland, Alice Burnett, Margaret Charlton, Marissa Clark, Noni Davis, Julianne Duff, Leah Hickey, Emily Johnston, Katherine Lee, Rheanna Mainzer, Marion McDonald, Bronwyn Novella, Gillian Opie, Lauren Pigdon, Gehan Roberts, Katherine Scott, Alicia Spittle, Penelope Stevens, Alice Stewart, Anne‐Marie Turner, Tania Woods

**Affiliations:** ^1^ Newborn Research Centre Royal Women's Hospital Melbourne Victoria Australia; ^2^ Department of Obstetrics and Gynaecology The University of Melbourne Melbourne Victoria Australia; ^3^ Murdoch Children's Research Institute Melbourne Victoria Australia; ^4^ Monash Newborn Monash Children' Hospital Melbourne Victoria Australia; ^5^ Ritchie Centre Hudson Institute of Medical Research Melbourne Victoria Australia; ^6^ Mercy Hospital for Women Melbourne Victoria Australia; ^7^ Turner Institute for Brain and Mental Health & School of Psychological Sciences Monash University Melbourne Victoria Australia

**Keywords:** development, prematurity, steroid

## Abstract

**Aim:**

Systemic postnatal corticosteroids are used to treat or prevent bronchopulmonary dysplasia (BPD) in extremely preterm (EP) or extremely low birth weight (ELBW) infants but are associated with long‐term harm. We aimed to assess the relationship between cumulative postnatal corticosteroid dose and neurodevelopmental outcomes.

**Methods:**

Longitudinal cohort study of all EP/ELBW livebirths in Victoria, Australia 2016–2017. Perinatal data were collected prospectively. Neurodevelopmental assessment was performed at 2 years' corrected age. Linear and logistic regression were used to determine relationships between cumulative corticosteroid dose and neurodevelopment, adjusted for gestational age, birth weight, sex and major intraventricular haemorrhage.

**Results:**

Seventy‐six EP/ELBW infants received postnatal corticosteroids to treat or prevent BPD, 62/65 survivors were seen at 2 years. Median (IQR) cumulative postnatal corticosteroid dose was 1.36 (0.92–3.45) mg/kg dexamethasone equivalent. Higher cumulative corticosteroid dose was associated with increased odds of cerebral palsy, adjusted OR (95% CI) 1.47 (1.04, 2.07). Higher cumulative corticosteroid dose was also associated with lower cognitive and motor developmental scores, however, this weakened after adjustment for confounding variables: cognitive composite score adjusted coefficient (95% CI) −1.3 (−2.7, 0.1) and motor composite score adjusted coefficient (95% CI) −1.3 (−2.8, 0.2).

**Conclusion:**

Higher cumulative postnatal corticosteroid dose in EP/ELBW infants is associated with increased odds of cerebral palsy at 2 years' corrected age. Adequately powered studies are needed to assess the independent effects of cumulative steroid dose on neurodevelopmental outcomes.

**Abbreviations**
BPD: bronchopulmonary dysplasia
CI: confidence interval
CP: cerebral palsy
ELBW: extremely low birth weight
EP: extremely preterm
GMFCS: gross motor function classification system
IQR: interquartile range
OR: odds ratio
SD: standard deviation
VICS: victorian infant collaborative study

**Key Notes**

- Although postnatal corticosteroids effectively treat bronchopulmonary dysplasia in extremely preterm or extremely low birthweight infants, they are associated with long‐term harm.
- Little is known about the association of higher cumulative postnatal steroid dose and long‐term outcomes.
- In this longitudinal cohort study of extremely preterm/extremely low birth weight infants, higher cumulative postnatal corticosteroid dose was associated with increased odds of cerebral palsy at 2 years' corrected age.

## 1 INTRODUCTION

Extremely preterm (EP, born <28 weeks' gestation) and extremely low birth weight (ELBW, birth weight < 1000 g) infants are at risk of bronchopulmonary dysplasia (BPD).^1^, ^2^ BPD, usually defined as the need for supplemental oxygen or respiratory support at 36 weeks' postmenstrual age,^3^ is a chronic lung disease that affects more than 50% of surviving EP infants.^1^ BPD is independently associated with higher mortality, increased incidence of cerebral palsy (CP) and higher rates of cognitive impairment.^4^, ^5^, ^6^, ^7^

Postnatal systemic corticosteroids are used to treat or prevent BPD in EP infants.^8^ Corticosteroids facilitate weaning from mechanical ventilation.^9^ However, some studies suggest that the neonatal administration of systemic corticosteroids may increase the risk of CP and neurodevelopmental disability in childhood, particularly when given in the first week after birth.^9^, ^10^ There are large variations between studies in the type, timing and dose of systemic corticosteroids used,^11^, ^12^, ^13^ and clinical practice also varies. A common treatment regimen follows the ‘DART’ protocol that administered a total of 0.89 mg/kg of dexamethasone, tapered over 10 days.^12^ However, it is common for EP/ELBW infants to receive doses that exceed those in the ‘DART’ protocol, or to receive more than one course.

The balance between the shorter‐term benefits of postnatal systemic corticosteroids versus the longer‐term risks is complex, and the safest and most effective postnatal corticosteroid regimen remains unclear. Furthermore, the relationship between cumulative postnatal corticosteroid dose and long‐term neurodevelopmental outcomes is not well understood.

This observational study in EP or ELBW infants aimed to determine the association between the cumulative postnatal corticosteroid dose given to treat or prevent BPD and neurodevelopment at 2 years of age (corrected for prematurity).

## 2 PATIENTS AND METHODS

This is a sub‐study of the Victorian Infant Collaborative Study (VICS) 2016/2017 cohort. VICS is a state‐wide collaboration of four tertiary‐level neonatal units (The Royal Women's Hospital, Monash Children's Hospital, the Mercy Hospital for Women and the Royal Children's Hospital) in Victoria, Australia. Live born EP (born <28 weeks' gestation) or ELBW (birth weight < 1000 g) infants, who were free of lethal anomalies, in the state of Victoria, Australia between 1st April 2016 and 31st March 2017 were recruited.^14^ The studies were approved by the Human Research Ethics Committees at all four centres. Written informed consent was obtained from all parents.

### 2.1 Perinatal data collection

All maternal, perinatal and neonatal data were collected prospectively. BPD was defined as supplemental oxygen dependency at 36 weeks' postmenstrual age. Postnatal corticosteroid use was defined as any systemic corticosteroids (either dexamethasone or hydrocortisone). The total cumulative dose of corticosteroid was calculated in milligrams per kilogram (mg/kg) dexamethasone equivalent, using the most recent infant weight available (usually recorded within the previous 3–7 days). Where corticosteroid courses were first administered with no corresponding weight recorded, either birth weight (if corticosteroid administration was within first 2 weeks after birth) or the closest recorded weight (administration after the first 2 weeks of life) was used. To standardise corticosteroid dosing, the cumulative dose of hydrocortisone was converted to a dexamethasone equivalent by dividing the cumulative hydrocortisone dose by a factor of 26.7.^15^ For the purpose of this analysis, infants who received ≤5 doses of corticosteroid were excluded, as these brief courses were likely prescribed for treatment of hypotension or upper airway obstruction, and not for BPD.

### 2.2 Outcomes at 2 years of age (corrected for prematurity)

At 2 years' corrected age, children were assessed by trained assessors blinded to clinical history and gestation at birth, for presence and severity of CP, blindness (visual acuity <6/60 in the better eye), deafness (hearing loss requiring amplification or a cochlear implant, or worse) and developmental delay. CP was determined by abnormal tone and reflexes, and a loss of motor function, with severity determined by the Gross Motor Function Classification System (GMFCS).^16^ Moderate–severe CP was defined as GMFCS level 2–5. Cognitive, language and motor development were assessed with the Bayley Scales of Infant and Toddler Development, 3rd edition (Bayley‐III).^17^ Major developmental delay was defined as greater than 2 standard deviations (SD) below the mean of term‐born controls for either the cognitive of language composite scores on the Bayley‐III. A child who was unable to complete the psychological testing because of severe developmental delay was assigned a score of −4 SD. Major neurodevelopmental disability was defined as the presence of any one or more of: moderate–severe CP, blindness, deafness or major developmental delay.^14^

### 2.3 Data extraction for this study

All EP/ELBW VICS 2016/2017 cohort participants were included in this study if they were offered intensive care. Data pertaining to the use of postnatal corticosteroids, respiratory outcomes and other important in hospital outcomes, were extracted from the VICS 2016/2017 dataset in addition to the 2‐year corrected age outcome data.

### 2.4 Statistical analysis

Data were analysed using STATA v17 (StataCorp). Participant characteristics were summarised using means (SD) for normally distributed continuous data, medians (interquartile ranges, IQR) for skewed continuous data and number and proportion (percent) for categorical data. To explore the associations between postnatal corticosteroid cumulative dose and outcomes, linear or logistic regressions were performed, fitted using generalised estimating equations and reported with robust (sandwich) estimates of standard errors to account for non‐independence of outcomes related to multiple births within the same family.

**FIGURE 1:**
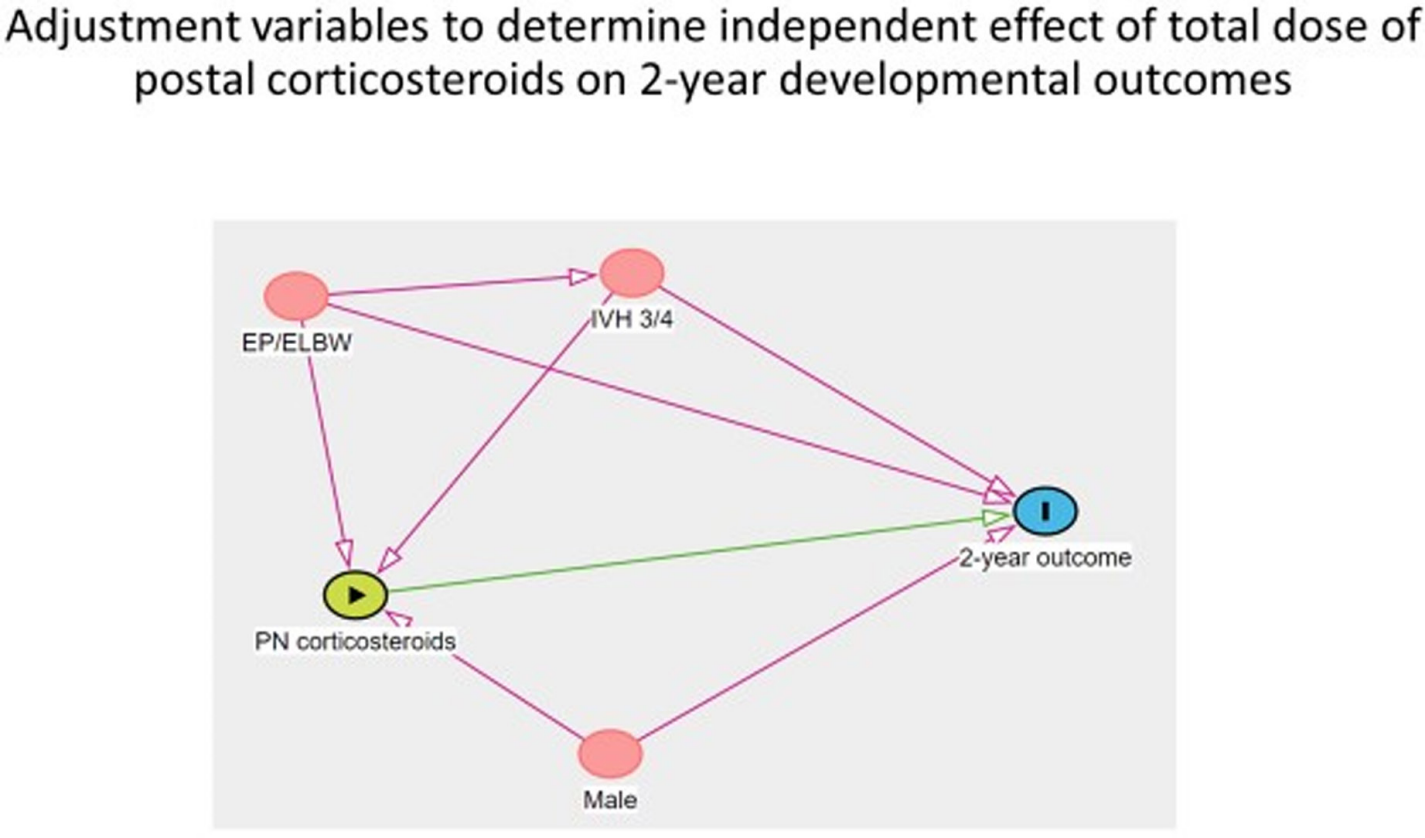
Directed acyclic graph for 2‐year developmental outcomes depicting assumed causal relationships between variables. ELBW, extremely low birthweight; EP, extremely preterm; IVH 3/4, intraventricular haemorrhagehemorrhage grade 3 or 4.

The analyses were performed firstly unadjusted, and then adjusted for potential confounding perinatal characteristics (gestational age at birth, sex, birth weight and major (Grade 3 or 4) intraventricular haemorrhage). Adjustments were based on a directed acyclic graph in order to identify the possible confounding variables (Figure 1). Associations were reported as coefficients or odds ratios (ORs) with 95% confidence intervals (CIs). We acknowledge multiple comparisons and focus on the overall strength of the evidence rather than presenting *p*‐values.

## 3 RESULTS

**FIGURE 2:**
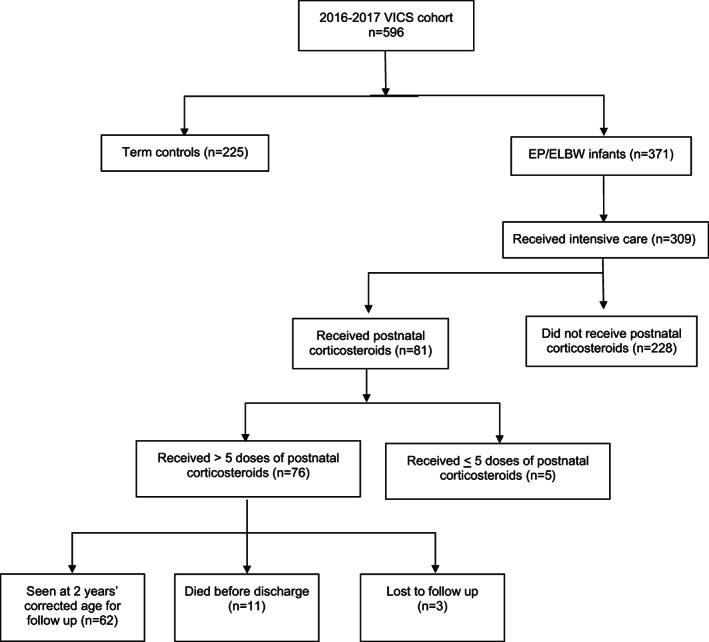
Eligibility flowchart.

There were 309 EP/ELBW infants who were offered intensive care. Within this group of EP/ELBW infants, 76 (24.6%) infants received systemic postnatal corticosteroids for the prevention or treatment of BPD and 62/65 (95.3%) of those who were alive at 2 years were assessed (Figure 2). Forty‐four (57.9%) infants received dexamethasone, 25 (32.9%) infants received hydrocortisone and 7 (9.2%) infants received both.

Infants in the ‘steroid’ group were more immature (mean 24.9 vs. 26.7 weeks' gestation) and had a lower birth weight (mean 720 vs. 879 g) than infants in the ‘no‐steroid’ group (Table 1). There was a higher proportion of males in the ‘steroid’ group (63.2% vs. 48.9%). In the steroid group, the median (IQR) cumulative postnatal corticosteroid dose was 1.36 (0.92–3.45) mg/kg of dexamethasone equivalent, the duration of corticosteroid treatment was 17.5 (11.0–38.5) days and the number of steroid doses received was 46 (20–103).

**TABLE 1:** Participant characteristics.

Variable	Steroid group (*N* = 76)	No steroid group (*N* = 233)
Maternal age (years), mean (SD)	32.4 (4.8)	32.0 (6.1)
Preeclampsia	12 (15.8)	33 (14.4) *n* = 229
Antenatal corticosteroids	68 (89.5)	202 (87.5) *n* = 231
Caesarean section	47 (61.8)	158 (67.8)
Multiple birth	22 (29.0)	69 (29.7)
Gestational age (completed weeks), mean (SD)	24.9 (1.4)	26.7 (1.9)
Male	48 (63.2)	114 (48.9)
Birth weight (g), mean (SD)	720 (130)	879 (181)
Birth weight *z*‐score, mean (SD)	−0.29 (1.03)	−0.48 (1.28) *n* = 232
Apgar score at 5 min, median (IQR)	7 (5, 8)	8 (6, 9)
Major intraventricular haemorrhage Grade 3 or 4	11 (14.5)	17 (7.4) *n* = 231
Cystic periventricular leukomalacia	2 (2.6)	2 (0.9) *n* = 232
In oxygen at 36 weeks' postmenstrual age	61 (87.1) *n* = 70	66 (32.0) *n* = 206
Two years		
Alive at 2 years	65 (85.5)	206 (88.4)
Seen at 2 years^a^	62 (95.4)	156 (75.7)
Cerebral palsy^b^	8 (12.9)	5 (3.3) *n* = 153
Blindness^b^	0 (0)	1 (0.6)
Deafness^b^	2 (3.2)	1 (0.6)
Major developmental delay^b^	21 (33.9)	27 (18.1) *n* = 149
Major disability^b^	21 (33.9)	17 (11.5) *n* = 148

*Note*: All data are *n* (%) unless specified.

^a^ % of those alive at 2 years.

^b^ % of those seen at 2 years.

Table 2 summarises the associations between cumulative postnatal corticosteroid dose (as dexamethasone equivalent) and 2‐year neurodevelopment. Although higher cumulative postnatal corticosteroid doses were associated with lower cognitive, language and motor development, these associations weakened following adjustment for gestational age, birth weight, sex and intraventricular haemorrhage. Higher cumulative doses of postnatal corticosteroids were associated with higher odds of CP at 2 years, a relationship that strengthened after adjustment: adjusted OR (95% CI) 1.47 (1.04, 2.07). There was little evidence for relationships between cumulative postnatal corticosteroid doses and major developmental delay and major disability at 2 years.

**TABLE 2:** Associations between cumulative dexamethasone equivalent dose and two‐year neurodevelopment.

Outcome variable	Unadjusted	Adjusted^a^
	Coefficient (95% CI), *p* value	
Cognitive composite score	−1.6 (−3.0, −0.2)	−1.3 (−2.7, 0.1)
Language composite score	−2.3 (−4.8, 0.3)	−1.6 (−3.9, 0.8)
Motor composite score	−1.7 (−3.3, −0.1)	−1.3 (−2.8, 0.2)
	**OR (95% CI), *p* value**	
Cerebral palsy	1.23 (0.94, 1.61)	1.47 (1.04, 2.07)
Major developmental delay	1.12 (0.87, 1.43)	1.10 (0.85, 1.44)
Major disability	1.16 (0.92, 1.45)	1.14 (0.91, 1.41)

*Note*: Coefficients represent change in outcome variable for each 1 mg/kg dose of dexamethasone received.

Abbreviations: CI, confidence interval; OR, odds ratio.

^a^ Adjusted for gestational age at birth, sex, birth weight and major intraventricular haemorrhage.

## 4 DISCUSSION

In a population cohort of infants born EP/ELBW, higher cumulative postnatal corticosteroid dose was independently associated with higher odds of CP, but not other domains of development. On average, EP/ELBW infants who were treated with postnatal corticosteroids in this study received a median dexamethasone equivalent dose equivalent to approximately 1.5 times the dose used in a single course in the DART trial.^12^ Approximately one‐quarter of infants received the equivalent of four or more DART courses and approximately one‐quarter received the equivalent of ≤1 course. While the mean reduction in Bayley scores were in the range of 1.6–2.3 points for each of the cognitive, language and motor domains, the confidence intervals extended to more than 4 points for the language domain which is a clinically important reduction in developmental scores.^17^ It is important to note that infants who received higher cumulative doses of postnatal corticosteroids were generally ‘sicker’ and thus were already at greater risk of adverse neurodevelopmental outcomes including cerebral palsy, developmental delay and major disability at 2 years' corrected age.

Onland et al.^18^ published a systematic review comparing systemic corticosteroid regimens for prevention of BPD in preterm infants. There were three categories of dexamethasone dosage regimens, that is, high (>4 mg/kg cumulative dose), moderate (between 2 and 4 mg/kg cumulative dose) and low (<2 mg/kg cumulative dose). Three studies compared a high dose to a moderate dose, and five studies compared a moderate dose to a low dose. The comparison between high and moderate dose favoured the higher dose regimen, with lower incidence of abnormal neurodevelopment. There was no difference in neurodevelopment when comparing the moderate to the lower dose regimens. The quality of evidence for these studies, however, was low to very low due to small number of events, publication bias and risk of performance, detection and attrition bias. Further, the dosages reflected the intended regimens rather than the actual dose of systemic corticosteroids that the individual infants would have received.

Another study on EP adolescents compared brain volumes at age 18 years in the EP group who had received postnatal corticosteroids (i.e. dexamethasone) to treat or prevent BPD in the newborn period with those who did not receive postnatal corticosteroids. Total brain tissue volumes, white matter, basal ganglia and thalami volumes were smaller in the postnatal corticosteroid group. Further, there was evidence for smaller total brain and white matter volumes with increasing cumulative dose of postnatal dexamethasone (regression coefficient − 7.7 [95% CI −16.2, 0.8] and −3.2 [−6.6, 0.2] per 1 mg/kg increase in dexamethasone cumulative dose respectively).^19^

The strengths of this study include the granularity of data available which enabled us to ascertain the true cumulative dose received by individual EP/ELBW infants rather than an intended dose regimen. The geographic nature of the cohort allows for generalisability of our findings. Follow‐up rates were high, with 95% of eligible infants assessed at 2 years. Follow‐up assessments were conducted by assessors blinded to clinical history, reducing bias. Limitations of the study include the observational nature rather than results reported from a randomised trial; thus limiting the ability to draw a causal relationship between postnatal corticosteroid dose and longer‐term neurodevelopmental outcomes. Despite adjustment, may have been additional sources of confounding that are not controlled for with this study design. The relatively small sample size limits interpretation and applicability of the results. Furthermore, the small sample size precluded separate analysis of infants who received hydrocortisone and those who received dexamethasone. Furthermore, assessments later in childhood may be required to reliably assess other neurodevelopmental outcomes.^20^

In clinical practice, deviations from ‘standard’ courses of postnatal corticosteroids are common, and in most cases are deemed necessary to save an infant's life or reduce the severity of BPD. The decision to administer doses of postnatal corticosteroids higher than the standard regimens should be taken with a clear understanding of the potential risks involved. These findings also justify further randomised trials with appropriate sample sizes and comparing different dosing regimens to explore the relationship between corticosteroid dose and long‐term outcomes.

In a cohort of EP/ELBW infants, we found an association between higher cumulative postnatal corticosteroid dose and greater risk of CP. This study indicates the need for caution in the use of higher doses of postnatal corticosteroids and prioritisation of longer‐term follow‐up of these infants.

## AUTHORS' CONTRIBUTIONS

ED was responsible for data collection and analysis, and co‐wrote the first draft of the manuscript. KH assisted with data collection, co‐wrote the first draft of the manuscript and then edited it. BJM, CTR, EJ, PA and PGD contributed to data collection and edited the manuscript. JO, LWD and JC assisted with data collection, data analysis and edited the manuscript. All authors approved the final version of the manuscript.

## FUNDING INFORMATION

Supported by grants from the National Health and Medical Research Council of Australia (Practitioner Fellowship #1157782 to PGD; Emerging Leadership Fellowship #1175634 to CTR; Leadership Fellowship), the Medical Research Future Fund of Australia (Career Development Fellowship #1141354 to JC and #1159225 to BJM) and the Victorian Government's Operational Infrastructure Support Program. The funding sources had no role in the study design; in the collection, analysis and interpretation of data; in the writing of the report and in the decision to submit the paper for publication.

## CONFLICT OF INTEREST STATEMENT

None.

## PRIOR PRESENTATION OF STUDY DATA AS AN ABSTRACT OR POSTER

Pediatric Academic Society Congress, Denver, Colorado, USA (April 2022). Perinatal Society of Australia and New Zealand Congress, Adelaide, Australia (May 2022).

## Data Availability

The deidentified data that support the findings of this study are available on request from the corresponding author. The data are not publicly available due to privacy or ethical restrictions

## References

[1] Chow SSW , Creighton P , Chambers GM , Lui K . Report of the Australian and New Zealand Neonatal Network 2020 . Sydney: ANZNN; 2022.

[2] Doyle LW , Spittle A , Anderson PJ , Cheong JLY . School‐aged neurodevelopmental outcomes for children born extremely preterm. Arch Dis Child. 2021;106(9):834‐838. doi:10.1136/archdischild-2021-321668 34035035

[3] Voynow JA . "New" bronchopulmonary dysplasia and chronic lung disease. Paediatr Respir Rev. 2017;24:17‐18. doi:10.1016/j.prrv.2017.06.006 28697967

[4] Cheong JLY , Doyle LW . An update on pulmonary and neurodevelopmental outcomes of bronchopulmonary dysplasia. Semin Perinatol. 2018;42(7):478‐484. doi:10.1053/j.semperi.2018.09.013 30401478

[5] Martin M , Smith L , Hofheimer JA , et al. Bronchopulmonary dysplasia and neurobehavioural outcomes at birth and 2 years in infants born before 30 weeks. Arch Dis Child Fetal Neonatal Ed. 2022. doi:10.1136/archdischild-2021-323405. Online ahead of print. PMC9947192 35999044

[6] Natarajan G , Pappas A , Shankaran S , et al. Outcomes of extremely low birth weight infants with bronchopulmonary dysplasia: impact of the physiologic definition. Early Hum Dev. 2012;88(7):509‐515. doi:10.1016/j.earlhumdev.2011.12.013 22236557 PMC3686277

[7] Doyle LW , Carse E , Adams AM , Ranganathan S , Opie G , Cheong JLY . Ventilation in extremely preterm infants and respiratory function at 8 years. N Engl J Med. 2017;377(4):329‐337. doi:10.1056/NEJMoa1700827 28745986

[8] Doyle LW . Postnatal corticosteroids to prevent or treat bronchopulmonary dysplasia. Neonatology. 2021;118(2):244‐251. doi:10.1159/000515950 33975319

[9] Doyle LW , Cheong JL , Hay S , Manley BJ , Halliday HL . Early (<7 days) systemic postnatal corticosteroids for prevention of bronchopulmonary dysplasia in preterm infants. Cochrane Database Syst Rev. 2021;10(10):Cd001146. doi:10.1002/14651858.CD001146.pub6 34674229 PMC8530019

[10] Doyle LW , Cheong JL , Hay S , Manley BJ , Halliday HL . Late (≥ 7 days) systemic postnatal corticosteroids for prevention of bronchopulmonary dysplasia in preterm infants. Cochrane Database Syst Rev. 2021;11(11):Cd001145. doi:10.1002/14651858.CD001145.pub5 34758507 PMC8580679

[11] Cummings JJ , D'Eugenio DB , Gross SJ . A controlled trial of dexamethasone in preterm infants at high risk for bronchopulmonary dysplasia. N Engl J Med. 1989;320(23):1505‐1510. doi:10.1056/nejm198906083202301 2657423

[12] Doyle LW , Davis PG , Morley CJ , McPhee A , Carlin JB . Low‐dose dexamethasone facilitates extubation among chronically ventilator‐dependent infants: a multicenter, international, randomized, controlled trial. Pediatrics. 2006;117(1):75‐83. doi:10.1542/peds.2004-2843 16396863

[13] Ramaswamy VV , Bandyopadhyay T , Nanda D , et al. Assessment of postnatal corticosteroids for the prevention of bronchopulmonary dysplasia in preterm neonates: a systematic review and network meta‐analysis. JAMA Pediatr. 2021;175(6):e206826. doi:10.1001/jamapediatrics.2020.6826 33720274 PMC7961472

[14] Cheong JLY , Olsen JE , Lee KJ , et al. Temporal trends in neurodevelopmental outcomes to 2 years after extremely preterm birth. JAMA Pediatr. 2021;175(10):1035‐1042. doi:10.1001/jamapediatrics.2021.2052 34279561 PMC8290336

[15] Singer MWA . Oxford Handbook of Critical Care. Oxford University Press; 2010.

[16] Palisano R , Rosenbaum P , Walter S , Russell D , Wood E , Galuppi B . Development and reliability of a system to classify gross motor function in children with cerebral palsy. Dev Med Child Neurol. 1997;39(4):214‐223. doi:10.1111/j.1469-8749.1997.tb07414.x 9183258

[17] Bayley N . Bayley Scales of Infant and Toddler Development. 3rd ed. Psychological Corp; 2005.

[18] Onland W , De Jaegere AP , Offringa M , van Kaam A . Systemic corticosteroid regimens for prevention of bronchopulmonary dysplasia in preterm infants. Cochrane Database Syst Rev. 2017;1(1):Cd010941. doi:10.1002/14651858.CD010941.pub2 28141913 PMC6464844

[19] Cheong JL , Burnett AC , Lee KJ , et al. Association between postnatal dexamethasone for treatment of bronchopulmonary dysplasia and brain volumes at adolescence in infants born very preterm. J Pediatr. 2014;164(4):737‐743.e1. doi:10.1016/j.jpeds.2013.10.083 24332820 PMC4029072

[20] Spencer‐Smith MM , Spittle AJ , Lee KJ , Doyle LW , Anderson PJ . Bayley‐III cognitive and language scales in preterm children. Pediatrics. 2015;135(5):e1258‐e1265. doi:10.1542/peds.2014-3039 25896835

